# Manual and Electroacupuncture for Labour Pain: Study Design of a Longitudinal Randomized Controlled Trial

**DOI:** 10.1155/2012/943198

**Published:** 2012-04-17

**Authors:** Linda Vixner, Lena B. Mårtensson, Elisabet Stener-Victorin, Erica Schytt

**Affiliations:** ^1^Division of Reproductive Health, Department of Women's and Children's Health, Karolinska Institutet, Retzius väg 13A, 171 77 Stockholm, Sweden; ^2^School of Health and Social Studies, Dalarna University, Högskolan Dalarna, 791 88 Falun, Sweden; ^3^School of Life Sciences, University of Skövde, P.O. Box 408, 541 28 Skövde, Sweden; ^4^College of Nursing, University of Rhode Island, White Hall, 2 Heathman Road, Kingston, RI 02881-2021, USA; ^5^Institute of Neuroscience and Physiology, Department of Physiology, Sahlgrenska Academy, University of Gothenburg, 405 30 Gothenburg, Sweden; ^6^Department of Obstetrics and Gynecology, First Affiliated Hospital, Heilongjiang University of Chinese Medicine, Harbin 150040, China; ^7^Centre for Clinical Research Dalarna, Nissers väg 3, 791 82 Falun, Sweden

## Abstract

*Introduction*. Results from previous studies on acupuncture for labour pain are contradictory and lack important information on methodology. However, studies indicate that acupuncture has a positive effect on women's experiences of labour pain. The aim of the present study was to evaluate the efficacy of two different acupuncture stimulations, manual or electrical stimulation, compared with standard care in the relief of labour pain as the primary outcome. This paper will present in-depth information on the design of the study, following the CONSORT and STRICTA recommendations. *Methods*. The study was designed as a randomized controlled trial based on western medical theories. Nulliparous women with normal pregnancies admitted to the delivery ward after a spontaneous onset of labour were randomly allocated into one of three groups: manual acupuncture, electroacupuncture, or standard care. Sample size calculation gave 101 women in each group, including a total of 303 women. A Visual Analogue Scale was used for assessing pain every 30 minutes for five hours and thereafter every hour until birth. Questionnaires were distributed before treatment, directly after the birth, and at one day and two months postpartum. Blood samples were collected before and after the first treatment. This trial is registered at ClinicalTrials.gov: NCT01197950.

## 1. Introduction

Methodology problems in acupuncture research have previously been highlighted [1], including the area of labour pain [2]. A recent systematic review [2] concludes that further high-quality studies are needed as the findings from existing studies [3–9] on the effect of acupuncture on labour pain are contradictory and difficult to interpret for a number of methodology reasons.

One such reason is that the studies have different primary outcomes. In total, seven randomized controlled trials (RCTs) were published in this area of research between 2002 and 2009. Of them, six trials report the women's subjective assessment of labour pain intensity as a primary outcome measure [4–9]. Two studies found that acupuncture was more effective than low-intensity “minimal acupuncture” at nonacupuncture points [6, 8]. On the other hand, three studies found no difference in pain intensity between women receiving acupuncture and women receiving standard care only [4, 7, 9] or with the addition of transcutaneous electrical nerve stimulation (TENS) [4] or administering acupuncture at nonacupuncture points [7]. A further study showed that sterile water injections reduced the intensity of labour pain more effectively than acupuncture [5] and that this effect is most likely mediated via similar mechanisms to high-intensity acupuncture (where needles are more frequently stimulated).

In two studies, the primary outcome was the use of pharmacological pain relief, where women who received acupuncture treatment used epidural analgesia and/or pethidine to a lesser extent than women who received standard care [3, 4] or with the addition of TENS [4]. However, this outcome measurement may be problematic as other factors influence the decision to use an epidural, such as the local culture at the delivery ward, the availability of an anaesthesiologist; or the woman's background [10]. In addition, the quality of midwife support influences the need of pharmacological pain relief [11]. As interventions such as acupuncture involve a high degree of midwifery presence, the possibility to draw proper conclusions from studies using such outcomes may be problematic. Other outcomes associated with conditions that may influence the experience of pain have also been evaluated but with contradictory results. In some studies, acupuncture increased the degree of relaxation [4, 9] while in others it did not [5, 7]. In some it shortened the time spent in labour [6, 8], and in others it did not [4, 5, 7, 9].

Another important methodological aspect is the variation in intensity of treatment between the studies. Acupuncture with frequent manual stimulation seems to have a better effect on labour pain than treatments with less stimulation, such as minimal acupuncture [6, 8]. Also, high-intensity treatments, such as sterile water injections, seem to relieve pain more effectively than low-intensity acupuncture [5]. Previous studies therefore raise important questions that need to be explored further regarding acupuncture treatment such as type of stimulation (electrical versus manual), number of needles used, stimulation intensity, and treatment duration.

In addition to this, most previously published studies include small sample sizes, and few include power calculations assuring that the study is large enough to detect relevant differences in outcomes. The treatment protocol (i.e., a precise description of the treatment including number of acupuncture points used, stimulation type, frequency of sessions, and length of treatment period) is often incomplete or absent, and does not follow the CONSORT [12] recommendations for reporting RCT nor the STRICTA document [13], which is a complement for reporting acupuncture studies specifically. Differences in content of care in the control group and the inclusion and exclusion criteria all make the results difficult to evaluate. Some studies give limited or no information on important factors such as possible maternal and neonatal side effects, timing of the treatment, intensity of the treatment, and training and skills of the personnel administering the treatment [3, 6, 7]. Women receiving treatment from a small group of midwives who were experienced in administering acupuncture seemed to benefit more from treatment [8] than women who were treated by a large number of midwives with varying training and experience [5]. Information on biological markers of pain and stress may further increase the understanding of possible effects of acupuncture in the relief of labour pain.

Acupuncture involves puncturing the skin with thin sterile needles at well-defined acupuncture points which may be excitable muscle/skin-nerve complexes with a high density of nerve endings [14]. The needles are primarily stimulated manually or electrically. In manual acupuncture (MA) the needles are twisted back and forth until a feeling of DeQi is reached. DeQi is described as a sensation of numbness, soreness, or heaviness reflecting the activation of afferent nerve fibres. The efficacy of the treatment depends on its intensity, which is related to the number of times DeQi is reached [15, 16]. In electroacupuncture (EA), needles are stimulated electrically [17]. Frequency and intensity are parameters known to influence the effect of EA.

The physiological mechanisms of acupuncture are not fully understood, but the pain-relieving effects may be explained by western medical theories [16]. Acupuncture needles activate receptors and afferent nerve fibres, in particular A*β*/*δ*-fibres and C-fibres. According to Melzack and Wall, the stimulation of A*β*-fibres activates the gate control mechanism that inhibits pain transmission at spinal level [18]. To activate these pain inhibiting systems, needles should be placed and stimulated in the same spinal segment as the source of the pain [19]. There are also extra segmental, central effects. Acupuncture triggers three main groups of opioid peptides, *β*-endorphin, enkephalin, and dynorphin [20]. Another effect of acupuncture is that it may activate another endorphin system—the diffuse noxious inhibitory Control (DNIC) system [21].

The nociceptive stimuli of the ripening and dilatation in the cervix in the initial stage of labour, known as the latent phase, are transmitted to the posterior root ganglia of thorasic spine Th10 to lumbar spine L1-L2. As the labour proceeds, more slowly in primiparous women than in multiparous, the nociceptive stimuli originate from the spinal segments S2–S4 [22, 23]. The nervous system, the immune system, and the endocrine system are all involved in the regulation of pain and the body's response to stress [24], and pain enhancement is mediated by glial activation and the release of proinflammatory cytokines [25]. Stressful experiences increase the circulation levels of proinflammatory cytokines [26], and possible associations between labour pain management, stress, and cytokines are underinvestigated.

Control interventions are a problem in acupuncture research [27] and the use of placebo has been discussed intensively [28, 29]. Placebo interventions include minimal or superficial acupuncture, needles on nonacupuncture points (sham acupuncture), or “placebo needles”, that is, needles with a handle that moves down over the needle, giving the false impression that they are inserted into the skin. A recent review demonstrates that placebo acupuncture differs from other physical and pharmacological placebo procedures in that it is associated with larger effects [30], possibly having a similar neurochemical basis with the activation of the endogenous opioid systems [19, 31], and sham acupuncture could be seen as a low-intensity form of therapeutic needling [27]. Consequently, placebo treatment does not seem to be a credible control intervention in acupuncture trials [19].

A review of previous studies indicates that acupuncture may have an effect on women's experiences of labour pain, but in order to enable a more definite conclusion further studies with better reporting to enable valid interpretations and replicability are needed.

## 2. Aim and Outcome Measurements

The aim of the present study was to evaluate the efficacy of two different acupuncture stimulations compared with standard care in the relief of labour pain. Our hypothesis is that acupuncture with manual or electrical stimulation is more effective than standard care in the relief of labour pain and that acupuncture with electrical stimulation is the most effective. This paper presents in-depth information on the design of the study and the ways in which we attempt to follow CONSORT [12] and STRICTA [13] recommendations.

### 2.1. Primary Outcome

Experience of labour pain.

### 2.2. Secondary Outcomes

Use of epidural analgesia.
Experience of relaxation.
Labour outcomes: mode of delivery, pain relief, augmentation of labour, duration of labour, and perineal trauma.
Negative side effects.
Experience of midwife support.
Proinflammatory cytokines, for example, interleukin (IL)-1, IL-6, highly sensitive C-reactive protein (hs-CRP), and tumor necrosis (TNF)-alpha.
Memory of labour pain and overall childbirth experience.
Infant outcomes: Apgar score, pH, BE, and neonatal transfer.

## 3. Materials and Methods

### 3.1. Design

**Figure 1:**
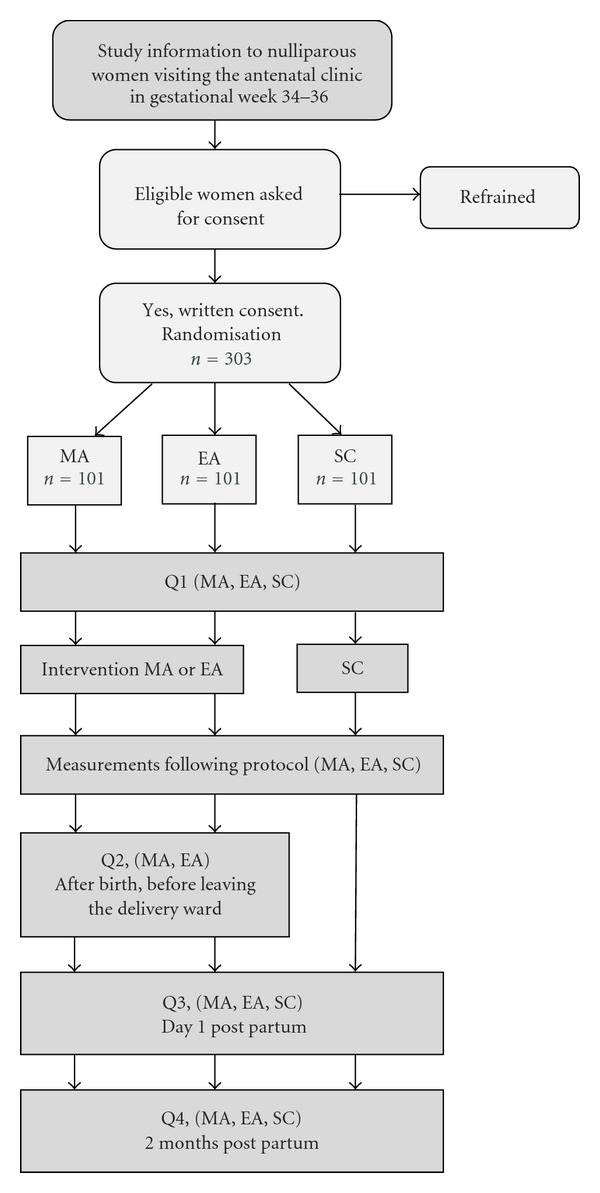
Study design including recruitment, randomization, interventions, and data collection. MA: Manual acupuncture, EA: Electroacupuncture, SC: Standard care, Q: Questionnaire.

The study was designed as a three-armed, randomized, and controlled trial in two delivery wards in two different hospitals in Sweden. A description of the study outline is presented in Figure 1. All eligible women who gave their written consent to participate in the study were randomly allocated to one of three groups: manual acupuncture (MA), electroacupuncture (EA), or standard care (SC). The rationale of acupuncture was based on Western medical theories, and the study protocol follows CONSORT [12] and STRICTA [13] recommendations. The study is registered at ClinicalTrials.gov: NCT01197950. Process evaluation was conducted by intermittent checkups in order to assure that the intervention procedures were performed correctly and that they followed study protocol.

### 3.2. Participants

All nulliparous women who were in gestational week 34–36 and attended regular checkups with a midwife at the antenatal clinics connected to the two hospitals received written and oral information about the study and an address to an informative study website (http://www.akupunkturstudien.se). Women were then asked to give consent to participate in the study when admitted to the labour ward.

#### 3.2.1. Inclusion Criteria

Spontaneous onset of labour.
Admission to the labour ward in latent or active phase of labour.
Nulliparity.
Singleton pregnancy, cephalic presentation.
Gestation: 37 + 0 to 41 + 6 (weeks + days).
Expressed need for labour pain relief.
Knowledge of the Swedish language good enough to understand written and oral instructions.

#### 3.2.2. Exclusion Criteria

Intake of pharmacological pain relief medication within 24 hours prior to inclusion into the study, with the exception of paracetamol.
Preeclampsia.
Treatment with oxytocin at the time point of allocation.
Treatment with anticoagulant.
Pacemaker.

After assessment of eligibility to the study, the women were randomized into one of the three groups. The randomization was conducted in blocks of 9, 12, and 15, which were varied randomly. A computerised random number generator generated a list of codes from 1 to 303, with each code linked to one of the three groups. Sequentially numbered, opaque, sealed envelopes were prepared by one of the authors (LV), which included a study protocol and four questionnaires. At the time of allocation, the assisting midwife picked the envelope with the lowest number on which she wrote the participant's name and social security number and then opened it.

### 3.3. Education of Midwives

The participating midwives had varying training and experience of administering acupuncture treatment (Table 1). We therefore conducted a one-day study-specific course, which included theoretical sessions with a Western medical approach to acupuncture physiology, practical sessions in MA and EA, and lectures on research methodology with a focus on RCT. The course was repeated once every semester. The midwives at the antenatal clinics also received in-depth information about the study and the mechanisms of acupuncture. Furthermore, all midwives had access to the open website and, with the use of a password, access to a closed section that included instructional videos and written information about the study.

### 3.4. Interventions

All women in the trial received care from midwives throughout labour and birth and from obstetricians in cases of deviation from normal progress, according to Swedish clinical practice. All participants had access to all pharmacological and nonpharmacological analgesia available in Swedish maternity care with the exception of women in the SC group who did not have access to any form of acupuncture.

A list of acupuncture points based on literature and in collaboration with experienced clinicians and tutors was established (Table 2). Local points were selected in muscle tissue in the pain area with the same somatic innervations as the cervix and uterus. In addition, points in hands and feet, so-called distal points, were selected in order to strengthen and prolong the effect of local needles. From the list, 3 bilateral distal points and 4–8 bilateral local points were chosen individually depending on pain location. In total, women in the MA and EA groups were treated with 13–21 needles. Sterile acupuncture Hegu Xeno needles for single use were used, sized 0.30 × 30 mm and 0.35 × 50 mm. In the MA group, all needles were stimulated until DeQi was reached every ten minutes for a 40-minute period. In the EA group, all needles were stimulated manually until DeQi was reached, and then eight of the local needles were connected to an electrostimulator (Cefar Acus 4, CEFAR, Lund, Sweden) which was set at high-frequency stimulation (80 Hz) square wave pulses (0.18 ms duration) with alternating polarity. The woman adjusted the intensity of the electrical stimulation so it was just under their pain threshold. The remaining needles were stimulated manually by the midwife until DeQi was reached every ten minutes for a 40-minute period. The needles were removed after 40 minutes in both groups. Two hours later the treatment was repeated. Additional treatment with MA or EA was thereafter available on request. After the first treatment with MA or EA, all women had access to standard forms of analgesia, if needed.

### 3.5. Data Collection

Established study protocols were filled in by midwives throughout the labour, including details on the labour, the intervention, and maternal and neonatal outcomes and by the women when making their assessments of labour pain and relaxation on VAS (Table 3). Data were also collected from patient records and by means of questionnaires (Table 4).

### 3.6. Measuring Pain and Relaxation

VAS was used for assessing experience of labour pain and relaxation. VAS is a 100 mm horizontal ungraded line with two endpoints: “no pain” (left) and “worst pain imaginable” (right) and “relaxed” (left) and “very tense” (right). Each woman assessed her level of pain/relaxation by making a vertical mark on the line with a sharp pencil [37]. VAS has been shown to be valid in detecting changes in pain intensity [38, 39] and relaxation [5, 7, 9, 40], and most individuals have no difficulties using it [38, 41]. One problem, however, is that the suitability of using VAS for monitoring labour pain has been questioned, as it has an apparent problem with response shift. As the labour proceeds, the pain intensity increases, and there is a possibility that the meaning of a value on VAS is changed (recalibrated) due to the higher pain intensity [42]. Although there are problems with using VAS to assess labour pain, it is currently the most common and best-validated pain scale used in research into labour pain. The measurements were conducted before the first treatment, immediately after the first treatment and then every 30 minutes for five hours and thereafter every hour until birth or until an epidural was administered. A different person from the one who administered the intervention (help nurse or midwife) assisted the women in the procedure of measuring pain and relaxation, however; blinding was not possible.

### 3.7. Questionnaires

Questionnaires were answered before the first treatment (Q1), immediately after birth (Q2), the day after birth (Q3), and two months later (Q4) (Table 4). Q1 asked about social-demographic background, previous experience of acupuncture, experience of menstrual pain, and how painful the woman expected the upcoming birth to be. Q2 was answered by women in the MA and EA groups only and included questions regarding experience of the acupuncture treatment, effects of the treatment, and negative side effects, if any. Q3 and Q4 were answered by all participants and asked about experience of labour [5, 34, 43–45], pain relief, memory of labour pain measured by VAS, experience of the intervention, support during labour, and emotional health problems in terms of depressive symptoms, which were assessed by the Edinburgh Postnatal Depression Scale [35, 36]. All protocols and questionnaires were pilot tested.

### 3.8. Blood Sampling

Blood samples were collected in the MA and EA groups before the first treatment and directly after the first treatment and in the SC group, before the first analgesic treatment and 30 minutes later. An indwelling intravenous catheter was inserted, and from this 5 mL of blood was drawn and collected in a standard tube. The samples clotted for a minimum of 30 minutes and no longer than four hours before they were centrifuged at 3000 rpm for 15 minutes at 20°C. Four polypropylene tubes of 0.5 mL with aliquots of serum and plasma were frozen at minus 70°C. Proinflammatory cytokines, for example, interleukin (IL)-1, IL-6, highly sensitive C-reactive protein (hs-CRP), and tumour necrosis (TNF)-alpha, will later be analysed by commercial assays at an accredited laboratory.

### 3.9. Sample Size Calculation

The sample size calculation was based on the primary outcome measure, experience of labour pain. The calculation used a Bonferroni adjusted significant level of 0.017 and a power of 0.80. A standard deviation of 20.4 mm on the VAS displayed in each group was brought from historical data [9]. To detect a difference of 15 mm on VAS between the three groups, 41 women in each group were needed. However, a previous study [5] shows that a high internal dropout could be expected because of labour-related factors, such as an inability to carry through the measurements due to pain or that birth has occurred. Two hours after the first treatment, only 47% of the women had registered data on pain or relaxation (personal communication Dr. L. B. Mårtensson January 2008). To compensate for this expected dropout rate, 88 women in each group were needed. Finally, another 15% dropout due to women's dissatisfaction with the randomization or midwives' high workload could be expected. This required a total of 101 women in each group, that is, 303 women in total.

### 3.10. Statistical Analyses

A problem with analyses of longitudinal data, is that repeated observations for the same individual are often correlated. This correlation violates the assumption of independence necessary for more-traditional, repeated-measures analysis and leads to bias in regression parameters. To avoid this problem we intend to use a mixed effects models approach in the analysis. Typically, ignoring the correlation of observations leads to smaller standard errors (SEs) and increases type I errors which might lead to the wrong conclusion [46, 47]. Furthermore, the analysis of mixed effect models enables to handle missing data as well as the integration of time-varying factors, such as cervical dilatation, which are issues in the present study.

## 4. Ethical Considerations

The study has no foreseeable risks but may cause minor discomfort in the form of tiredness or minor bruising. The women were informed that (1) participation in the study was voluntary, (2) their decision whether or not to participate would not affect their current or future treatment, (3) if they decided to participate they were free to withdraw at any time, and (4) all questionnaires and blood samples would be unidentified. The women who agreed to participate in the study signed a consent form. The study was approved by the Regional Ethical Review Board, University of Gothenburg, 2008-05-15, Dnr: 136-08.

## 5. Summary

Since the evidence of the effect of acupuncture on labour pain is nonconclusive or lacking, possibly because of methodological reasons, there is a need for more well-designed studies, and this study intends to fill such a gap by avoiding some of the limitations of previous research. Many women would like to have nonpharmacological pain relief during labour, and acupuncture is one such treatment that is available in all delivery units in Sweden. However, if acupuncture is not proven to be effective, it should not be recommended for labour pain, which is possibly the most intensive pain a woman may ever experience.

## Figures and Tables

**Table 1:** Participating midwives' previous education and experience with administrating acupuncture, prior to study training, *n* = 38.

	*n*
Education prior to study training	
*One day course with a midwife at the delivery ward*	10
Practical focus with acupuncture points chosen on TCM basis	
Included neither acupuncture physiology nor EA	
*Five-day course with focus on TCM and WM*	14
Practical and theoretical sessions.	
Acupuncture physiology based on both TCM and WM	
EA not included.	
*Six-day course in WM*	11
Practical and theoretical sessions	
Acupuncture physiology and research within the area of acupuncture and obstetrics	
EA included	
*No previous education in acupuncture at all*	3
Experience of administrating acupuncture during labour, prior to study	
None	12
<1 year	5
1-2 years	10
3–10 years	8
>10 years	3

TCM : traditional Chinese medicine, EA : electroacupuncture, WM : western medical acupuncture.

**Table 2:** Acupuncture points used in the study.

Points	Segmental innervation	Tissue	Depth (cun)
Distal points			

GV 20	Nn. trigeminus (V), occipitalis minor (C2), occipitalis major (C2-3)	Aponeurosis epicranii	0.3–0.5
LI 4	Nn. medianus ulnaris (C8-Th1)	Mm. interosseus dorsalis I, lumbricalis II, adductor pollicis	0.5–1
SP 6	N. tibialis (L4-S1)	Mm. flexor digitorum longus, tibialis posterior	0.5–1
LR 3	N. plantaris lateralis (S2-3)	M. interosseus dorsalis I	0.3–0.5
PC6	N. medianus (C8, Th1)	M. flexor digitorum superficialis	0.5–0.8
EX2	N. trigeminus	M. frontalis	0.3–0.5
LU7	N. cutaneus antebrachii lateralis (C5-6)	Fibrous tissue	0.3–0.5

Local points			

SP 12	N. thoracicus (Th7–12), lumbalis (L1)	Aponeurosis mm. obliquus externus, abdominis internus	0.5–1
BL 23	Nn. thoracodorsalis (C6–8), thoracicus (Th9–12), lumbalis (L1–3)	Mm. serratus posterior inferior, erector spinae, fascia thoracolumbalis	0.8–1
BL 24	Nn. thoracodorsalis (C6–8), thoracicus (Th9–12), lumbalis (L1–3)	Mm. erector spinae, fascia thoracolumbalis	0.8–1
BL 25	Nn. thoracodorsalis (C6-8), thoracicus (Th9–12), lumbalis (L1–3)	Mm. erector spinae, fascia thoracolumbalis	0.8–1
BL 26	Nn. thoracodorsalis (C6–8), thoracicus (Th9–12), lumbalis (L1–3)	Mm. erector spinae, fascia thoracolumbalis	0.8–1
BL 27	Nn. thoracodorsalis (C6–8), thoracicus (Th9–12), lumbalis (L1-3)	Mm. erector spinae, fascia thoracolumbalis	0.8–1
BL 28	Nn. thoracodorsalis (C6–8), thoracicus (Th9–12), lumbalis (L1–3)	Mm. erector spinae, fascia thoracolumbalis	0.8–1
BL 36	N. gluteus inferior (L5–S2)	M. gluteus maximus	1–1.5
BL 54	N. gluteus inferior	M. gluteus maximus	1.5–2
GB 25	N. thoracicus (Th7–12)	M. obliquus externus abdominis	0.3–0.5
GB 26	N. thoracicus (Th7–12)	M. obliquus externus abdominis	0.5–0.8
GB 27	Nn. thoracicus lumbalis (Th7–L1)	Aponeurosis mm. obliquus externus, internus abdominis	0.5–1
GB 28	M. obliquus externus abdominis	Aponeurosis mm. obliquus externus, internus abdominis	0.5–1
GB 29	N. gluteus superior (L4–S1)	M. tensor fasciae latae	0.5–1
LR 10	N. femoralis (L2-3)	M. pectineus	0.5–1
LR 11	N. femoralis (L2-3)	M. pectineus	0.5–1
KI 11	Nn. thoracicus (Th6–12), subcostalis (Th12)	Mm. pyramidalis, rectus abdominis. Vagina m. recti abdominis	0.5–1
ST 29	N. thoracicus (Th6–12)	M.rectus abdominis	0.7–1.2
CV3	N. iliohypogastric (L1)	Fibrous tissue	0.5–1
CV4	N. subcostalis (Th12)	Fibrous tissue	0.5–1

GV: governor vessel channel, LI: large intestine channel, SP: spleen channel, LR: liver channel, PC: pericardium channel, EX: extra channel, LU: lung channel, BL: bladder channel, GB: gall bladder channel, KI: kidney channel, ST: stomach channel, CV: conception Vessel, Cun: traditional Chinese unit of length, 1 cun: width of the distal interphalangeal joint of the thumb.

**Table 3:** Content of study protocol, similar for all groups.

Concept	Measurement and response alternatives
Mother	

Pain during contraction	VAS, every 30 minutes for 5 hours, and then every hour until birth
Relaxation during contraction	VAS, every 30 minutes for 5 hours, and then every hour until birth
Pain localisation	Back/Abdomen/Groin. every 30 minutes for 5 hours, and then every hour until birth
Cervix dilatation and length	Cm 3 times during 5 hours
Contractions (duration/interval)	Seconds/Minutes 5 times during 5 hours
Details of intervention	Point selection from Table 2, duration of treatment, stimulation technique and stimulation frequency
Additional pain relief	Sterile water injections/TENS/Entonox/Opioid epidural and intrathecal analgesia/Pudendal nerve block/Paracervical block/Other
Midwives's evaluation of the treatment effect for pain relief and relaxation (MA and EA)	Very effective/Fairly effective/Not so effective/Not effective at all
Negative side effects (EA, MA)	Yes, if so, a description of the side effects/No
Augmentation of labour	Yes, if so, indication primary and secondary dystocia, other/No
Rupture of membranes/Amniotomy	Date/Time
Partus	Date/Time
Mode of delivery	Vaginal delivery/Vacuum extraction/Forceps/Emergency caesarean section
Perineal injury	Degree I–IV

Infant	

Apgar score	1, 5 and 10 min
Birth weight	Grams
Arterial and venous blood gases	pH/Base Excess (umbilical cord samples)
Neonatal transfer	Yes/No

VAS: visual analogue scale, TENS: transcutaneous electrical nerve stimulation, MA: manual acupuncture, EA: electroacupuncture.

**Table 4:** Content of questionnaires, before treatment (Q1), and postnatal questionnaires (Q2–Q4).

Concept	Response alternative	Questionnaire
Previous acupuncture experience	Yes for pain/Yes for other than pain/No	Q1
Dysmenorrhea	Yes, if so, estimation on VAS/No	Q1
Worry of pain in daily life*	Not at all worried/Not very worried/Quite worried/Very worried	Q1
Worry of labour pain*	Not at all worried/Not very worried/Quite worried/Very worried	Q1
Sociodemographic background	Education/Ancestral homeland/Parents citizenship	Q1
Postnatal valuation of treatment effect on pain and relaxation (MA and EA)	Very effective/Rather effective/Not very effective/Not effective at all	Q2, Q3, Q4
Use this treatment again? (EA, MA)	Yes/No	Q2, Q3, Q4
Negative side effects (EA, MA)	Yes, if so, description of side effects/No	Q2, Q3, Q4
Prelabour worries for: (a) labour pain*, (b) not enough pain relief, (c) not enough support from midwife	Not at all worried/Not very worried/Quite worried/Very worried	Q3
Support from midwife during labour	Yes to a high extent/Yes to a rather high extent/No to a rather low extent/No not at all	Q3, Q4
Overall experience of pain during labour	VAS	Q3, Q4
Overall experience of relaxation during labour	VAS	Q3, Q4
Experienced labour pain in relation to expected**	Much more severe than expected/More severe than expected/As expected/Milder than expected/Much milder than expected	Q3, Q4
Assessment of midwife's acupuncture skills (EA, MA)	Very competent/Quite competent/Not very competent/Not competent at all	Q3, Q4
Overall assessment of pain relief	Very effective/Rather effective/Not very effective/Not effective at all	Q3, Q4
Sufficiency of pain relief	Enough/Not enough	Q3, Q4
Emotions during labour*	Strong/Weak/Happy/Sad/Calm/ Frightened/Alert/ Tired/Secure/Worried/Involved/Lonely/ Detached/Independent/Empowered/ Abandoned/Determined/Tense/Trust in my own capacity/Challenged/Focused/Panic/Disappointed/Present	Q3, Q4
Emotions during labour, overall	Positive/Negative	Q3, Q4
Perception of the midwife*	Calm/Rushed/Supportive/Unhelpful/Clear/ Incompetent/Rude/Humorous/Inconsiderate/Sensitive/ Bossy/Absent/Warm/Nonchalant/Secure/Condescending/ Considerate/Competent/Vague/Informative/ Insensitive/Supportive	Q3, Q4
Overall perception of the midwife	Positive/Negative	Q3, Q4
Why participate in this study?	Open-ended	Q3
Satisfaction with the allocation.	Yes satisfied/No, if so, which allocation would you have preferred?	Q3
Overall birth experience^†^	Very positive/Positive/Mixed feelings/Negative/Very negative	Q3, Q4
Depressive symptoms	Edinburgh Postnatal Depression Scale^††^	Q4
Experience of participating in this study	Positive/Negative	Q4

Q1–4: questionnaire 1–4, VAS: visual analogue scale, MA: manual acupuncture, EA: electroacupuncture.

*Schytt et al. [32], adjusted for this study, **Experience of pregnancy and delivery, the women's perspective [33], ^†^Waldenström [34], ^††^Wickberg and Hwang [35], and Murray and Carothers [36].
